# A 53-year-old female with a large breast sarcoma: A case report from Hong Kong^☆^

**DOI:** 10.1016/j.radcr.2022.05.051

**Published:** 2022-06-21

**Authors:** Max K.H. Yam

**Affiliations:** North District Hospital, 9 Po Kin Road, Sheung shui, the New Territories, Hong Kong

**Keywords:** Breast sarcoma, Breast cancer, Mastectomy

## Abstract

Breast sarcomas are very rare and are aggressive tumors associated with a poor prognosis. We present a case of a 53-year-old female who presented to the hospital after noticing a palpable mass in the right breast. Mammography, ultrasound, and MRI investigations were done. The patient was treated with radical mastectomy combined with both neoadjuvant and adjuvant chemoradiotherapy. Undifferentiated pleomorphic breast sarcoma was later diagnosed.

## Introduction

Breast sarcoma is a rare but aggressive entity, accounting for less than 1% of all breast cancer cases, and core biopsy is required for diagnosis [1]. Tumors typically spread through local invasion or hematogenous spread [2]. Breast sarcoma typically affects patients aged 55-59 years [3]. Based on the clinical and imaging results, it is difficult to differentiate breast sarcoma from other types of breast cancer [4].

## Case report

A 53-year-old female was admitted to the hospital due to a bloody discharge from the right nipple. A physical examination revealed a large, firm, and fixed mass in her right breast.

**Fig. 1:**
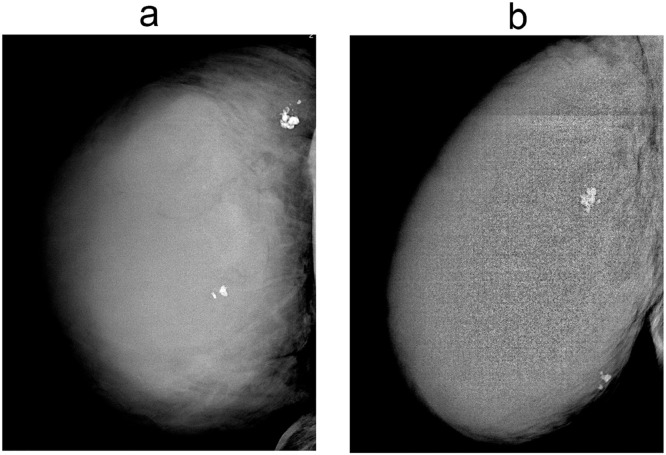
Craniocaudal view (Left) and mediolateral oblique view (Right) of a mammogram. A high-density mass in the right breast.

The mammogram showed breast asymmetry with a large high-density mass occupying the right breast. It was associated with architectural distortion and skin thickening (Fig. 1).

**Fig. 2:**
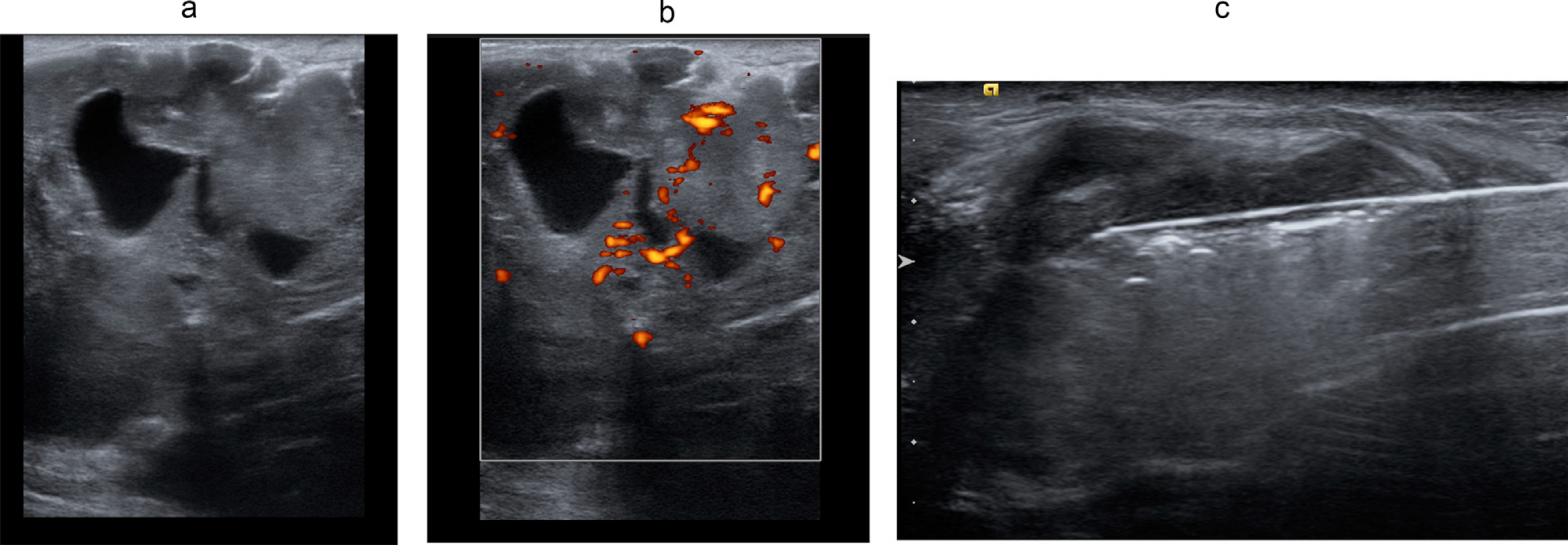
Ultrasound images of the right breast. A huge heterogeneous mass in the right breast (Left), with increased vascularity (Middle) and internal cystic areas. The border is circumscribed. Ultrasound-guided core biopsy performed using a 14G Maxcore needle (Right).

Subsequent breast ultrasound revealed a large, heterogeneous echotexture located in the right breast. It had increased vascularity and internal cystic areas. The border was relatively circumscribed. US-guided core biopsy was performed using a 14G Maxcore needle with local anesthesia (Fig. 2).

**Fig. 3:**
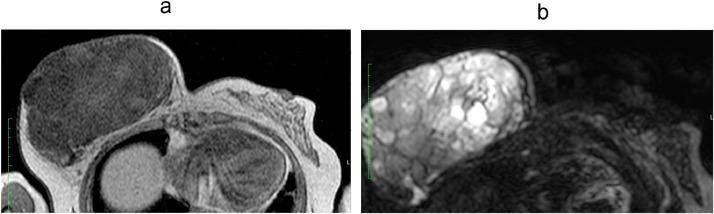
There is a huge contrast enhancing lesion with a circumscribed margin measuring 17.4 × 10.2 × 18 cm (TSxAPxCC) occupying the right breast. It appears isointense on T1-weighted image (Left) with areas of hemorrhage and heterogeneous signal on T2-weighted image (Right) with areas of cystic change.

Subsequent magnetic resonance imaging (MRI) revealed that the mass of the right breast was a huge contrast enhancing lesion with a circumscribed margin measuring 17.4 × 10.2 × 18 cm (TSxAPxCC). It appeared to be isointense on T1-weighted images with areas of hemorrhage and heterogeneous signal on T2-weighted images with areas of cystic change (Fig. 3).

**Fig. 4:**
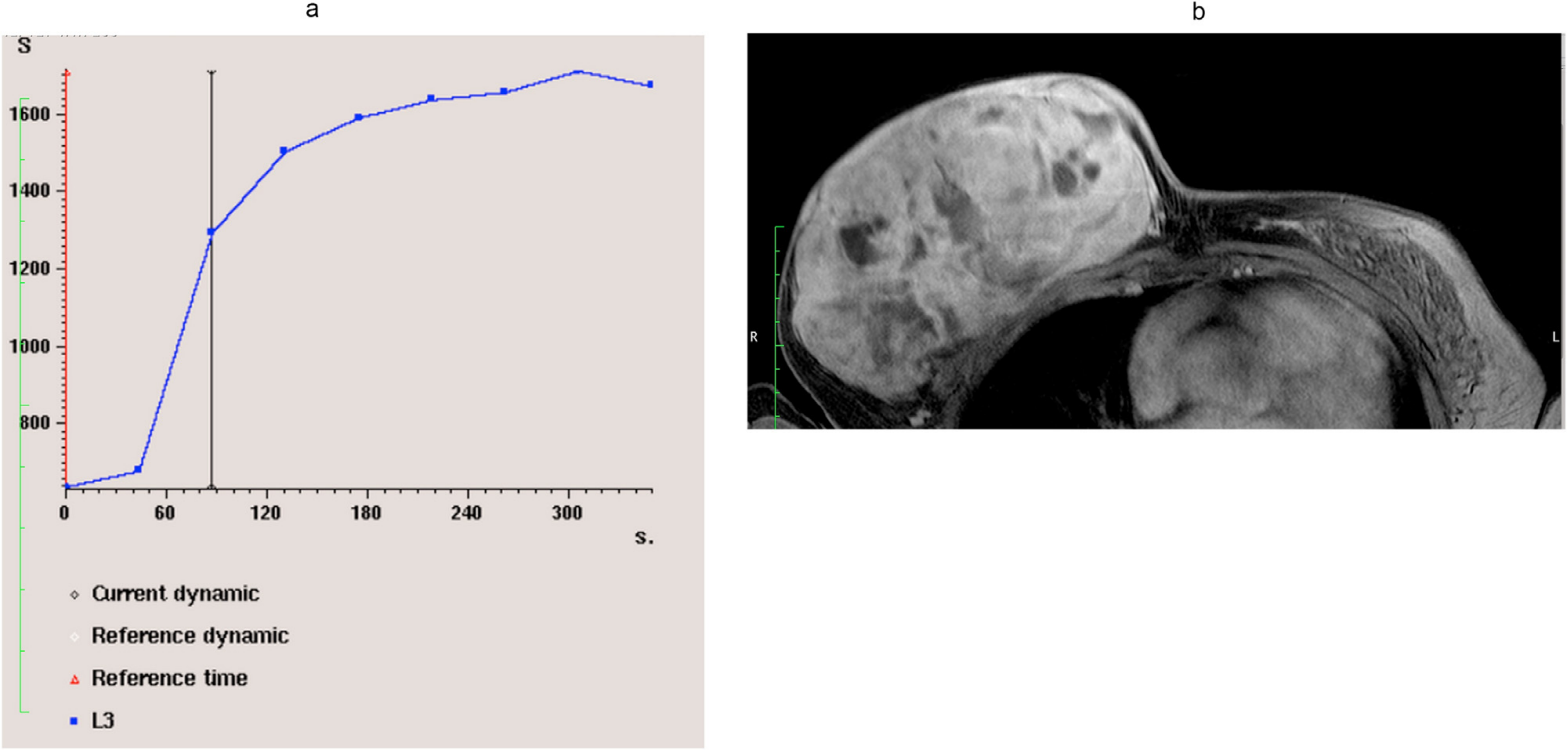
Diffusion-weighted image (Right) and ADC map (Left) showed areas of restricted diffusion.

**Fig. 5:**
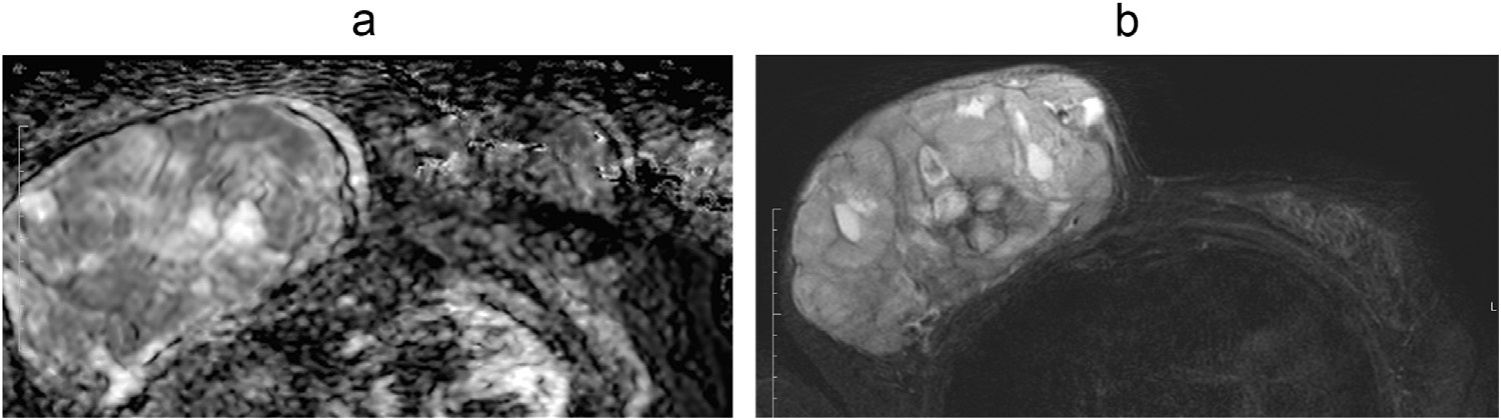
Dynamic postcontrast scan showed areas of early arterial enhancement (Right). Type II (plateau) kinetic curve (Left).

DWI showed areas of restricted diffusion (Fig. 4). Dynamic post contrast scan showed areas of early arterial enhancement in the superior anterior aspect with a type II (plateau) kinetic curve (Fig. 5). Features were suspicious of malignant lesions. There was however no definite evidence of dermal, underlying pectoralis muscle or nipple invasion.

A few prominent to mildly enlarged (up to 1.3 × 1.8 cm) were noted in the right axilla; nodal involvement could not be excluded.

Histological examination revealed the tumor to be composed of malignant cells that are spindled, with extensive areas of necrosis and hemorrhage, and other areas were vascular. Mitoses were numerous. In the absence of morphological and evidence of epithelial differentiation within the extensively sampled tumor, the diagnosis of metaplastic carcinoma or malignant phyllodes tumor were unlikely. A diagnosis of undifferentiated pleomorphic sarcoma was made.

The patient was treated with radical mastectomy combined with both neoadjuvant and adjuvant chemoradiotherapy. Unfortunately, the patient enjoyed a year-long disease-free survival before emigration to the Philippines and was unable to follow-up after one disease-free year.

## Discussion

Breast sarcomas are rare with an annual incidence of 4.6 cases in 1,000,000 women, representing less than 1% of all breast malignancies. These tumors can be primary or secondary (after previous treatment for breast cancer). There are several histological subtypes, the most common of them being angiosarcoma and malignant phyllodes tumor.

Breast ultrasound, mammography or MRI can be useful. These investigations may provide information regarding local aggressiveness, current lymph node status.

The tumors usually present as an oval hyperdense tissue that can be either circumscribed or indistinct, without calcification, on mammography [5]. Most primary breast sarcomas at sonography are oval solid hypoechoic masses with posterior acoustic enhancement that may mimic phyllodes tumors. In the appropriate clinical context, breast radiologists should be alerted of this overlap in imaging features.

The tumors are commonly present as hyperintense on T2-weighted images on MRI, with indistinct margins and heterogeneous enhancement [6].

Immunohistochemistry may be useful for distinguishing breast sarcomas from nonmesenchymal malignant tumors. In the present case, the final diagnosis was made by excluding other breast cancer types.

Due to the rarity and heterogeneity of this type of neoplasm, the treatment is not well established. Depending on the tumor stage, preoperative chemotherapy, radiotherapy, and chemoradiation, associated with surgery and adjuvant chemotherapy, are recommended for soft-tissue sarcoma treatment [7].

Breast sarcomas have a high recurrence rate and poor prognosis [8]. The overall 5-year survival rate is approximately 50% in patients with undifferentiated pleomorphic sarcoma [9].
